# Obituary: pulmonary artery catheter 1970 to 2013

**DOI:** 10.1186/2110-5820-3-38

**Published:** 2013-11-28

**Authors:** Paul E Marik

**Affiliations:** 1 Division of Pulmonary and Critical Care Medicine, Eastern Virginia Medical School, 825 Fairfax Avenue, Suite 410, Norfolk, VA, USA

**Keywords:** Pulmonary artery catheter, Right heart catheterization, ICU, Hemodynamic monitoring, Operating room

## Abstract

The birth of the intermittent injectate-based conventional pulmonary artery catheter (fondly nicknamed PAC) was proudly announced in the *New England Journal of Medicine* in 1970 by his parents HJ Swan and William Ganz. PAC grew rapidly, reaching manhood in 1986 where, in the US, he was shown to influence the management of over 40% of all ICU patients. His reputation, however, was tarnished in 1996 when Connors and colleagues suggested that he harmed patients. This was followed by randomized controlled trials demonstrating he was of little use. Furthermore, reports surfaced suggesting that he was unreliable and inaccurate. It also became clear that he was poorly understood and misinterpreted. Pretty soon after that, a posse of rivals (bedside echocardiography, pulse contour technology) moved into the neighborhood and claimed they could assess cardiac output more easily, less invasively and no less reliably. To make matter worse, dynamic assessment of fluid responsiveness (pulse pressure variation, stroke volume variation and leg raising) made a mockery of his ‘wedge’ pressure. While a handful of die-hard followers continued to promote his mission, the last few years of his existence were spent as a castaway until his death in 2013. His cousin (the continuous cardiac output PAC) continues to eke a living mostly in cardiac surgery patients who need central access anyway. This paper reviews the rise and fall of the conventional PAC.

## Review

Pulmonary artery (PA) catheterization was first performed by Lewis Dexter in 1945 [1]. After observing a spinnaker on a sailboat off Santa Monica beach, the idea of a flow-directed PA catheter (PAC) was developed by Swan and Ganz in 1970, allowing bedside placement [2]. The PAC was subsequently modified with a thermistor to allow measurement of cardiac output (CO) [3]. Shortly after the publication by Swan *et al*. in 1970, the balloon tipped PA catheter became commercially available and it began to be used in a variety of clinical settings. The use of the PAC moved from the cardiac catheterization laboratory to the ICU and operating room and its use changed from being used as a diagnostic to a therapeutic tool. Clinicians began to use the hemodynamic data derived from the PAC to select, modify and monitor medical treatments. After the introduction of the PAC, enthusiasm for the device increased and its use increased exponentially. Indeed, the PAC became the cornerstone of critical care and a hallmark of the ICU. In the 1980s 20% to 40% of seriously ill patients who were hospitalized were reported to undergo pulmonary artery catheterization [4]. This phenomenon occurred despite that fact that the safety, accuracy, and benefit of the device had never been established.

Eugene Robin was the first clinician to challenge the widespread adoption of the PAC. He wrote two editorials in the mid 1980s in which he called for a moratorium on the use of the PAC until randomized controlled trials (RCTs) were performed which demonstrated the safety and improved outcomes associated with the use of the PAC [5][6]. A decade later the landmark study by Connors and coworkers was published [7]. Using propensity matching, this study demonstrated a 24% increased risk of death in ICU patients who received a PAC within 24 hours of admission to an ICU. The first large randomized, controlled, prospective, evaluation of the PAC was published by Sandham and colleagues in 2003 [8]. These authors randomized 1,994 high risk patients aged 60 years or older who were scheduled for major surgery to goal directed therapy guided by a PAC compared to standard care without a PAC. Hospital and six month mortality and length of stay were similar between the two groups. Except for pulmonary embolism, which was higher in the PAC group, morbidity was similar between groups. This was followed by two European studies conducted in critically ill ICU patients and the ESCAPE trial in patients with heart failure which failed to demonstrate any benefit from the PAC [9][10][11]. A meta-analysis conducted by the Cochrane group demonstrated no benefit from the use of the PAC in high risk surgery patients (eight studies) and general ICU patients (four studies) [12]. In the US, the use of the PAC peaked between 1993 and 1996 with a rate of 5.6 per 1,000 hospital admissions, declining to a rate of 1.99 per 1,000 hospital admissions in 2004 [4]. The decline in the use of the PAC appears to have followed the publication of the Connors study in 1996 [7]. Current utilization of the PAC in ICU’s in the US is unknown, with most PACs being placed in patients undergoing cardiothoracic surgery. Despite dramatic reductions in the routine use of the PAC in cardiac surgery to less than 20% of cases in Europe and less than 10% in Japan, some centers in North America and Australia still routinely use the device [13][14].

The PAC provides hemodynamic data that cannot be obtained from clinical examination. It was therefore widely believed that the data obtained from the PAC would allow a better understanding of the patients hemodynamic profile and that this would lead to therapeutic interventions which would improve patient outcomes [15]. This premise was based on the assumption that the data obtained from the PAC were accurate, that clinicians were able to interpret the data and that the data themselves were useful in managing critically ill patients. However the evidence suggests that these three assumptions are incorrect. Furthermore, it is likely that information obtained from the PAC triggers inappropriate therapy that may be harmful.

### The data provided by the PAC may not be accurate

The CO and pulmonary capillary wedge pressure (PCWP) are the hemodynamic parameters obtained from the PAC which are most frequently used to make therapeutic decisions. Furthermore, these variables are used in the calculation of many of the other hemodynamic parameters. However, the inaccuracies of both of these variables essentially preclude them from being used for this purpose.

Adolph Fick described the first method of CO estimation in 1870 [16]. The direct Fick method was the reference standard by which all other methods of determining CO were evaluated until the introduction of the PAC. Currently the PAC is considered the ‘Gold Standard’ against which other devices are compared. Despite the ubiquitous use of the PAC, remarkably few studies have investigated the accuracy of the CO measurements as determined by thermodilution. In 1982 Stetz and colleagues analyzed the reliability of the PAC and reported a precision of 15%; that is, there must be a minimal difference of 15% between determinations of CO (three measurements per determination) to suggest clinical significance [17]. This study became the reference standard for the PAC, with a precision of 15% being used in studies evaluating fluid responsiveness. However, more recent studies have been unable to reproduce the findings of Stetz and colleagues. Dhingra and coauthors compared the thermodilution CO with that measured by the Fick technique over a wide range of cardiac outputs [18]. The bias was −0.17 L/min with the upper and lower limits of agreement being 2.96 L/min and −3.30 L/min, respectively. The percentage error was 62%. When compared to the direct Fick method the PAC has a percentage error of 56 to 83% [18][19][20]. The percentage error (2 SD/μ) is derived by the Bland-Altman method with a percentage error of up to 30% being considered clinically acceptable [21]. Philips *et al*. compared thermodilution CO with surgically implanted ultrasonic flow probes in an ovine model [22]. The percentage bias and precision was −17% and 47% respectively; the PAC under-measured dobutamine-induced CO changes by 20% (relative 66%) compared with the flow probe. This study found that the PAC was an inaccurate measure of CO and was unreliable for detection of CO changes less than 30%. Critchely *et al*., using a similar methodology in pigs, reported a precision of 26% [23]. These studies suggest that the true CO has to change by at least 25% to be detected by the PAC. Furthermore, the required change may be as high as 100% depending on the monitor being used [24].

It is likely that multiple factors interact to affect the accuracy of the thermodilution CO calculation [25]. Occult warming of cold indicator before injection can produce indicator loses leading to overestimates of CO. Significant losses of thermal indicator arise from the dissipation of cold indicator through the intravascular portions of the catheter which have been pre-warmed by the surrounding blood. Spontaneous or mechanical ventilation affects the actual CO; the SV can vary by as much as 50% at various phases of the respiratory cycle [26][27]. The averaging of multiple measurements at different phases of the respiratory cycle has therefore been proposed. It is unclear how many measurements are needed for sufficient accuracy and reproducibility, but it seems that three, although clinically mostly performed, is insufficient. PA thermodilution CO measurements are unreliable in the presence of tricuspid regurgitation. In general CO is underestimated in patients with tricuspid regurgitation [28][29][30]. This finding is important as the incidence of tricuspid regurgitation is about 15% in the general population increasing to greater than 70% in elderly patients [31][32][33]. It should be noted that the reproducibility of CO measurements by transpulmonary thermodilution appears significantly better than that of the PAC with a precision of about 7% [34][35]. The better precision of transpulmonary thermodilution compared to the PAC is probably related to the longer transit time of the thermal bolus which is not influenced by respiration and arrhythmias.

In addition to providing CO data that are inaccurate, a number of factors may lead to erroneous PCWP measurements. The accurate and consistent placement of pressure transducers for invasive monitoring is critically important. Errors in zeroing and obtaining baseline measurements are exceedingly common and may result in changes in the PA and PCWP pressure of up to 6 mmHg [36][37]. Furthermore the patient’s position is frequently not standardized, leading to further errors in measurement [38]. Figg and Nemergut demonstrated significant variation in transducer placement for central venous pressure (CVP) measurement amongst perioperative health care providers [39]. Damped tracings and catheter ‘fling’ may not be recognized, leading to erroneous measurements [38]. These errors are compounded in patients on mechanical ventilators where the use of positive pressure ventilation, spontaneous breaths and the use of positive end expiratory pressure (PEEP) make analysis of the PCWP challenging and unreliable [36][40][41].

### Risks from the PAC itself

The complications that may arise directly from the use of the PAC include pulmonary artery rupture, pulmonary artery thrombosis, intra-cardiac knotting of the catheter, pulmonary hemorrhage, right atrial thrombosis, catheter related bloodstream infection, internal jugular/subclavian vein stenosis or thrombosis, atrial and ventricular arrhythmia, electromechanical dissociation and right-sided endocarditis. These risks may not be trivial. A study of 70 critically ill patients demonstrated that 4% died from complications related to the PAC and that between 20 and 30% had major complications related to the PAC [42]. Fatal air embolism related to the PAC introducer has also been reported [43][44][45].

### Harm due to knowledge deficit

Studies suggest that clinicians are unable to correctly interpret the data obtained from the PAC even if one assumes that these measurements are accurate. A 1990 study by Iberti *et al.*, in which a 31-item examination on the PAC was completed by 496 North American ‘intensivists’ found that only 67% of the answers were correct [46]. The instrument yielded similar results in Europe [47]. A 1996 survey of more than 1,000 critical care physicians found that, although 83% of questions were answered correctly, a third of the respondents could not correctly identify the PCWP on a clear tracing and could not identify the major components of oxygen transport [48]. Large interobserver variability has been reported in the interpretation of PAC pressure tracings with little agreement between ‘experts’ [49][50][51]. A survey of practicing cardiac anesthesiologists concluded that ‘a large proportion of anesthesiologists who use the PAC disagree about PCWP estimation, and even those who agree may lack the confidence necessary to use it effectively’ [52]. What is most disturbing is that when board certified intensivists are provided with the same PAC data there is enormous variability in the intervention consequent to ‘interpretation’ of the data [51]. Remarkably, while clinicians acknowledge that ‘other practitioners’ have a poor understanding on the PAC and the interpretation of the ‘hemodynamic profile’ derived from the PAC, they believe that they have a good understanding of the PAC and that in their ‘hands’ the PAC is a useful and beneficial device [51][52][53]. Similar problems concerning a ‘knowledge deficit’ have been identified among critical care nurses [54]. Johnson and colleagues using the same questionnaire as Iberti *et al*. in a cohort of ICU nurses demonstrated only 42% of questions were answered correctly [55]. In this study, 51% of respondents were unable to correctly identify the pressure change as the catheter was advanced from the right ventricle to the PA. It is clear that this ‘knowledge deficit’ is a major factor contributing to the lack of benefit (? harm) of the PAC.

### The data obtained from the PAC may not be useful in managing critically ill patients

A major factor explaining the lack of benefit of the PAC may be that the data obtained are not useful in managing critically ill patients. A commonly cited benefit of the PAC is that it provides filling pressures which can be used to identify fluid responsiveness and guide fluid administration [15]. However, these filing pressures have been found to be neither uniformly accurate nor effective for fluid guidance. The PCWP suffers from the same limitation as the central venous pressure (CVP) [56][57]. Multiple studies have shown a poor relationship between the PCWP and circulating blood volume, SV and left ventricular end-diastolic volume [58][59][60][61][62][63]. Furthermore, the PCWP is unable to predict fluid responsiveness [64][65][66]. These data indicate that the PCWP should not be used to make decisions regarding fluid management. Furthermore, due to the inherent inaccuracy of the CO measurement the change in the SV following a fluid bolus cannot be used to determine fluid responsiveness nor construct a Frank-Starling curve. An increase in SV of 10 to 15% is used to define fluid responsiveness [66]. This threshold is significantly below the ability of the PAC to detect a change in CO [22]. In addition to its inability to determine fluid responsiveness the CO itself has very little utility in guiding patient management. Attempts at increasing CO and/or achieving supra-normal levels of oxygen delivery in medical patients have universally failed to positively impact patient outcome [9][12][67]. Indeed, the study by Hayes and colleagues demonstrated that such an approach is harmful [68]. ‘Paradoxically’, Morelli *et al*. demonstrated that in the setting of septic shock, the use of a beta-blocker which reduced CO and oxygen delivery improved patient survival [69].

### The PAC results in ‘overtreatment’

Survey studies in postoperative and ICU patients have demonstrated that the PAC provided ‘new information’ or seemed to change therapy in 30 to 62% of cases [70][71][72]. However, the clinical significance of these changes is uncertain and in the absence of demonstrated benefit it is likely that many of these interventions were not beneficial. Fellahi *et al*. demonstrated a significant independent increase in cardiac morbidity and in-hospital mortality in cardiac surgical patients who received dobutamine to improve CO based on PAC values [73]. Sandison *et al*. compared the outcome of patients undergoing non-elective abdominal aortic aneurysm repair in two different centers [74]. In the one center, PACs were inserted in 96% of cases versus 18% in the other. The patients in the center with higher PAC usage received more crystalloid, colloid and inotropes. Their incidence of renal failure was noted to be higher as were their lengths of stay in both ICU and hospital. These data suggest that placement of a PAC may result in excessive and inappropriate therapeutic interventions that have the potential to harm patients.

### Potential benefits of the PAC

The benefits of the PAC are somewhat difficult to define. In some patients the diagnosis between non-cardiogenic and cardiogenic pulmonary edema is difficult to make. In such circumstance the PCWP has been used to make this distinction. However, with advancements in echocardiography, catheterization of the pulmonary artery for this purpose is seldom required. It would appear that the role of the PAC is limited to diagnosing patients with pulmonary hypertension and managing these patients in the perioperative period (see below), as well as the diagnosis of intracardiac shunts (echocardiography may be better) and amniotic fluid embolism [75].

#### Indications for the use of the PAC

Doppler echocardiography is frequently used to calculate the pulmonary artery systolic pressure (sPAP). However, the sPAP as determined by Doppler echocardiography is an inaccurate estimate of sPAP, [76][77] and this technology is not considered a reliable method for the diagnosis and management of pulmonary arterial hypertension (PAH) [78]. Pulmonary artery catheterization is therefore required to confirm the diagnosis of PAH, classify PAH, assess its severity and to test the vasoreactivity of the pulmonary circulation [79][80]. Pulmonary artery catheterization has been recommended in patients with significant PAH (sPAP > 50 mmHg and/or RV enlargement) undergoing a major surgical intervention [81]. While this recommendation is not supported by high quality evidence, it would appear to be logical as the PA pressure is the only reliable hemodynamic parameter derived from the PAC and its use may allow for the rational titration of vasoactive agents.

Over 30 randomized controlled trials have studied perioperative hemodynamic optimization in a variety of settings, using various goals and techniques of hemodynamic optimization [82][83][84]. This approach has been demonstrated to reduce the risk of complications and mortality in elective non-cardiac surgery patients [82][83][84]. The initial preemptive hemodynamic studies used the PAC and targeted ‘supranormal’ goals while more recent studies have ‘optimized’ CO using esophageal Doppler or dynamic indices of fluid responsiveness. Meta-analyses of these studies have demonstrated that both approaches reduce surgical mortality and morbidity [82][83][84]. In addition, these meta-analyses have demonstrated that the PAC has been largely replaced by less invasive hemodynamic monitoring techniques.

## Conclusions

There is no evidence that the use of the PAC has improved patient outcomes. There are, however, convincing data that the hemodynamic parameters obtained from the PAC are inaccurate, are incorrectly interpreted and that these data frequently lead to excessive and inappropriate therapeutic interventions that maybe harmful. In addition, the data suggest that the hemodynamic parameters obtained from the PAC have little utility in managing critically ill patients in the ICU and operating room. These data therefore suggest that the PAC has a limited role in the ICU and operating room and challenge those experts who believe that the ‘*pulmonary artery catheter is still a valuable tool for hemodynamic monitoring*’ [85]. The PAC, however, has a role in the diagnosis and operative management of patients with pulmonary hypertension and acute right ventricular failure.

## Abbreviations

PCWP: Pulmonary capillary wedge pressure; CO: Cardiac output; PAC: Pulmonary artery catheter; RCT: Randomized controlled trial; PA: Pulmonary artery; CVP: Central venous pressure; PEEP: Positive end expiratory pressure; SV: Stroke volume; sPAP: Pulmonary artery systolic pressure; PAH: Pulmonary artery hypertension; RV: Right ventricle.

## Competing interests

The author has no financial interest in any of the products mentioned in this paper.
